# Polyacrylamide Hydrogel Containing Starch and Sugarcane Bagasse Ash: Synthesis, Characterisation, and Application in Cement Pastes and Mortars

**DOI:** 10.3390/ma17235889

**Published:** 2024-12-01

**Authors:** Ana Elizabete Nunes Pereira, Edson Araujo de Almeida, Fábio Rodrigo Kruger, Edson Cavalcanti da Silva-Filho, Edvani Curti Muniz

**Affiliations:** 1 Chemistry Postgraduation Program, Federal University of Piaui—UFPI, Teresina 64049-550, PI, Brazil; anaelizabete.np@gmail.com (A.E.N.P.); edsonfilho@ufpi.edu.br (E.C.d.S.-F.); 2 Chemistry Postgraduation Program, State University of Maringa—UEM, Maringá 87020-900, PR, Brazil; edsonalmeida2009@gmail.com; 3 Civil Construction Department, Federal University of Technology-Parana—UTFPR-CM, Campo Mourão 87301-889, PR, Brazil; fabiorodrigo_civil@hotmail.com; 4 Materials Science Postgraduation Program, Federal University of Technology-Parana—UTFPR-LD, Londrina 86036-370, PR, Brazil

**Keywords:** hydrogel, starch, sugarcane bagasse, hydrolysis, mortar, mechanical resistance

## Abstract

Internal curing is a process based on the addition of materials that function as water reservoirs in cementitious media. Superabsorbent hydrogels are an alternative that can be used as an internal curing agent, as they have the ability to absorb and release water in a controlled manner. In the present work, superabsorbent hydrogels based on crosslinked polyacrylamide in the presence of starch and sugarcane bagasse ash (SCBA) were developed and applied to mortars as an internal curing agent. The synthesized hydrogels were evaluated by SEM, FTIR, and swelling analysis. Cement pastes and mortars were produced using different amounts of hydrogel (0.03%, 0.06%, and 0.1% by weight). An analysis of the cement pastes and mortars revealed that hydrogel contributes to hydration, thus improving the quality of the product. Furthermore, the addition of 0.03% hydrogel by weight increased the mechanical resistance of the mortars in up to 26.8% at 28 days of curing as compared with reference (without hydrogel). To the best of our knowledge, this is the first study to use a hydrogel based on polyacrylamide crosslinked with starch and SCBA as a curing agent for mortars and cement pastes. This approach is environmentally friendly, because it uses a natural product (starch) and a byproduct from the sugarcane industry (SCBA).

## 1. Introduction

In recent decades, there has been a marked growth in both civil construction and the trailer industry. These changes have demanded new types of cementitious materials that meet different requests in current and unconventional applications [1]. One of the most used materials in this field is cement, which can be utilised in the production of concrete and mortars [2]. Mortars are heterogeneous composite materials used in civil construction; they comprise a binder (cement, lime, or gypsum), fine aggregate (sand), and water. Their use is versatile: they can serve as binding elements for other materials (blocks, bricks, and stones) and be used as surface coverings, such as plaster and base for ceramic coverings [3].

When a cementitious material comes into contact with water, hydration reactions begin and underlie the curing process. Proper mortar curing requires maintaining a satisfactory moisture content and temperature in freshly cast mortar for a specific period of time immediately following placement [4]. An effective curing process can drastically reduce the appearance of fissures and cracks [5]. So, it is important to maintain constant moisture in the mix during mortar curing, which minimises the effect of water loss through evaporation. Curing also helps maintain heat in the mixture, which speeds up the chemical reaction between cement and water [6]. In the case of cement, the cementitious mixture is moistened by the presence (or further introduction) of water for the hydration reactions. The main product of cement hydration is calcium silicate hydrate (C-S-H). Poorly cured concrete loses around 30% of its strength, thus facilitating the appearance of fissures and cracks in its structure [7].

There has been much attention directed towards the curing process due to the technological importance of cementitious materials, including their expected long life and the safety of their use [8]. Currently, internal curing is gaining prominence as an alternative to improve the hydration of the cementitious environment [9]. Internal curing involves supplying water to the aggregates in the cementitious material, which can occur by incorporating a curing agent into the cement and/or mortars. In other words, as the hydration reactions take place, the internal curing agent releases water in a controlled manner into the cementitious matrix [9,10]. The released water is concomitantly used in the chemical reactions that comprise the curing process.

One of the options to increase the water concentration in a cementitious material is the application of superabsorbent hydrogels [11]. Hydrogels are networks of crosslinked polymers that can absorb large amounts of water and release the absorbed water in a gradated manner over time. The water inside the hydrogel structure allows for the diffusion of the solute through the swollen polymer, allowing it to be applied as a release system control [12,13,14]. In this sense, hydrogels participate in the controlled release of water and can improve the continuation of hydration reactions that occur over time in the cementitious matrix, thus ensuring a more satisfactory internal cure [3]. Hydrogels can be synthesised from different materials, using synthetic ones, such as acrylamide/acrylate monomers, and/or natural polymers, such as polysaccharides [15]. A good example of a natural polymer used in hydrogel formulations is starch due to its composition, structure, and great abundance [15,16]. Starch is a complex polysaccharide composed of two components, amylose and amylopectin, each of which presents a specific characteristic structure [15,16]. Starch appears in a considerable proportion in plants, including cereal grains (rice, corn, etc.) and tubers (potatoes, cassava, etc.). So, it is easy to acquire in natural or chemically modified form [17], and it is often not expensive. Therefore, starch is one of the important natural polymeric materials [16,17].

Hydrogels prepared through the combination of natural and synthetic moieties have been described in the literature [15,18] and represent a good alternative for several technological applications [13,15,19,20,21]. The swelling process of a hydrogel is guided by physical factors related to the three-dimensional (3D) network, such as the degree of crosslinking and the presence of hydrophilic groups (e.g., -COOH, -OH, and-NH_2_) in the structure of the polymeric chains, and by external factors, such as the temperature, pH, and ionic strength [12,20,21]. The presence of hydrophilic groups on polymeric chains contributes to lowering the required crosslinking density and allows for high flexibility of the polymeric network [22]. In this sense, low crosslinking and high chain flexibility certainly contribute to the swelling of the studied material. The incorporation of sugarcane bagasse ash (SCBA), a burned agro-industrial residue, into the formulations for a cementitious matrix is another alternative to improve the properties of cement-based materials (i.e., concrete and mortars). The amount of silica in SCBA can facilitate curing and might improve the mechanical properties of the prepared concrete and mortar [23,24].

This work presents a new approach to prepare hydrogels, namely polymerisation/crosslinking of acrylamide in the presence of starch and SCBA. The hypothesis is that applying such a hydrogel in mortar and concrete improves the mechanical properties [23,24]. So, mortar formulations with different hydrogel content (0.03, 0.06, and 0.10 by weight) were prepared. The prepared hydrogels were characterised with scanning electron microscopy, Fourier transform infrared (FTIR) spectroscopy, and water absorption/desorption analysis, and the mortars were characterised by FTIR spectroscopy, X-ray diffraction (XRD), thermogravimetric analysis (TGA), and compressive strength assays.

## 2. Materials and Methods

### 2.1. Materials

Acrylamide (AAm, Sigma-Aldrich, St. Louis, MO, USA) was used as a monomer in colourless solid form with a melting point of 80 °C, boiling point of 125 °C, and density of 1.12 g/cm^3^; potassium persulfate (Synth, São Paulo, Brazil) in white powder form, with a melting point of 100 °C and density of 2.477 g/cm^3^, was used an as initiator; and *N,N’*-methylenebisacrylamide (MBAAm, Fisher Scientific, Hampton, NH, USA) in white powder form, with a melting point above 300 °C, boiling point of 333.8 °C, and density of 1.216 g/cm^3^, at 20 °C was used as a crosslinking agent. The hydrogels also comprise commercial cassava starch (dissolved in distilled water) and SCBA; the properties of SCBA may vary depending on the conditions in which the sugar cane was grown. The SCB was purchased locally in Teresina–Piauí, Brazil and then burned at a specific temperature at atmospheric pressure to obtain SCBA. The mortars were prepared with Portland cement type CP II-F and sand (normal Brazilian in four fractions: coarse (1.19 mm), medium coarse (0.59 mm), medium fine (0.297 mm), and thin (0.149 mm), according to the ABNT/NBR 7214/2015 [25].

### 2.2. Methods

#### 2.2.1. Preparation of SCBA, Synthesis, and Hydrolysis of the Hydrogels

The SCBA was obtained by burning SCB in an EDG-3000 muffle furnace at 600 °C for 2 h. Subsequently, the ash was crushed and sieved (75 mesh) [1]. The hydrogels were prepared by using a 2^3^ factorial design with three replications at the central point, totalling 11 formulations. The influence of three factors—(i) the amount of acrylamide, (ii) the amount of starch, and (iii) the amount of SCBA—(shown in Table 1) on the swelling capacity of the hydrogels was analysed. The levels for each factor were defined based on studies that have already synthesized acrylamide hydrogels in the presence of rice husk ash [26]. From this factorial design (shown in Table 2), the hydrogels with the highest swelling values were chosen for the subsequent experiments. 

To prepare the hydrogels, initially, the starch was dissolved in distilled water (using sufficient volume to maintain the concentration of the entire formulation at 136 g L^−1^) at 50 °C and magnetically stirred until its complete solubilization. Then, the solution was stirred for 24 h at room temperature. After this process, the required amount of SCBA was added, keeping the system at 70 °C with magnetic stirring. After 10 min, the required amount of AAm, 0.04 g of MBAAm, as a crosslinker, and 0.08 g of potassium persulfate, as an initiator, were added, with 10 min between the addition of each reactant. The system was stirred until it gelled. The hydrogels were cut into small pieces and immersed in distilled water (ca. 1 g swollen hydrogel per 50 mL of water) for washing. The water was changed every hour for 6 h. Then, the washed material was placed in an oven at 60 °C for 24 h to dry. After drying, the hydrogels were crushed and sieved (250 mesh).

For the hydrolysis of the hydrogels, 40 mL of 0.5 mol L^−1^ NaOH solution was added per 1 g of dry hydrogel. In a hood, the solution was magnetically stirred for 1 h at 50 °C. Then, the hydrogels were washed with distilled water until reaching pH 7 and, subsequently, dried in an oven at 60 °C for 24 h [27].

#### 2.2.2. Preparation of Mortars and Cement Pastes

**Figure 1:**
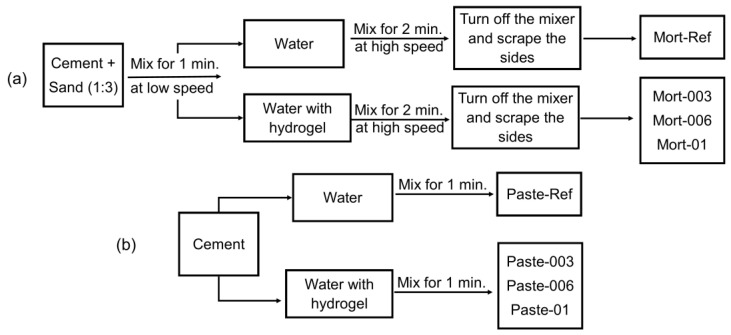
Procedure for preparing the (**a**) mortar and (**b**) cement paste formulations.

The hydrolysed H2 hydrogel (Table 2) was applied to the mortar formulations at 0.03%, 0.06%, or 0.1% relative to the mass of the cement (*w*/*w*). The hydrolysed H2 hydrogel was chosen due to its degree of swelling (Q) of 200.20 g/g. The mortars were prepared in accordance with ABNT/NBR 7215 standards [28]. The cement/sand ratio was 1:3. As described in Section 2.1, four fractions of sand were used. The hydrogels were crushed, sieved (250 mesh), and swollen over a period of 20 h. The cement pastes were produced by applying the same amount of hydrolysed H2 hydrogel used in mortars, with the aim of better understanding the hydration process, because they are less complex than mortars, as they only contain cement hydration products [29,30]. Figure 1 shows the preparation of the mortars and cement pastes.

### 2.3. Characterisation

#### 2.3.1. Degree of Swelling and Desorption of Hydrogels in Aqueous Solutions

The water absorption of the prepared hydrogels over time was measured using solutions at pH 4, 7, and 11 and in saline solution (0.1 mol L^−1^ NaCl). For these measurements, approximately 10 mg of dry hydrogel was used. Each hydrogel was immersed in 100 mL of solution and weighed with an analytical balance after 1, 2, 3, 4, 5, 6, 7, 8, 24, 48, and 72 h. The degree of swelling (Q) was calculated using Equation (1):

Q = [(m − m_0_)/m_0_] (1)

where m is the mass of the swollen hydrogel, and m_0_ is the dry mass of the hydrogel sample. To measure desorption, the hydrogels were swollen to equilibrium in distilled water and then transferred to an oven at 40 °C. The mass was determined after 1, 2, 3, 4, 5, 6, and 24 h. The results are represented as the percentage of remaining water, with the initial mass of the swollen hydrogel considered to be 100% water. The swelling and desorption tests were carried out in triplicate.

#### 2.3.2. Fourier Transform Infrared Spectroscopy (FTIR)

The hydrogel samples were dried and crushed with 250 mesh. Attenuated total reflectance (ATR)-FTIR spectra were obtained using a Vertex 70 apparatus (Bruker, Billerica, MA, USA) in the range of 600–4000 cm^−1^ for the hydrogels and the range of 400–4000 cm^−1^ for the mortars. The mortars and cement pastes were aged 7 and 28 days and then dried and crushed. Pastilles were prepared in the form of potassium bromide tablets at 1% (*w*/*w*).

#### 2.3.3. X-Ray Diffraction (XRD)

X-ray diffractograms of the mortars, in powder form, for 2(θ) of 10°−70° were obtained using an XRD-6000 diffractometer (Shimadzu, Kyoto, Japan) operating at a speed of 1° min^−1^, a divergent angle of 1°, 40 kV Cu radiation, and a current of 30 mA.

#### 2.3.4. Thermogravimetric (TGA)

Thermogravimetric was performed to monitor the degree of hydration and curing reactions of mortars aged for 28 days. A DTG-60 apparatus (Shimadzu) was used with nitrogen as a carrier gas, a temperature of 25–600 °C, a heating rate of 10 °C, and a flow rate of 100 mL min^−1^.

#### 2.3.5. Scanning Electron Microscopy (SEM)

A Quanta 250 scanning electron microscope (FEI) was used to analyse the morphology of the hydrogels. The samples were covered with a thin layer of gold (ca. 50 nm). The microscope operated at an accelerating voltage of 20 kV. The pore size and its distribution were measured using Image J^®^ 1.8.0 software; for this, the pore contours were manually delimited through the SEM images.

#### 2.3.6. Compressive Strength Tests

The compressive strength tests of the mortars were performed according to the NBR 7215 standard (ABNT/1996) [28,31], using cylindrical specimens with 50 mm in diameter and 100 mm in height. The samples were aged for 7 and 28 days and analyzed in quadruplicate in an EMIC Pc 100 machine, applying a load of 0.3–0.8 MPa s^−1^ until rupture. The tests provided the force applied to the specimen. The individual strength was calculated in mega Pascals (MPa) as a function of the specimen cross area.

## 3. Results

### 3.1. Water Absorption of Hydrogels

**Figure 2:**
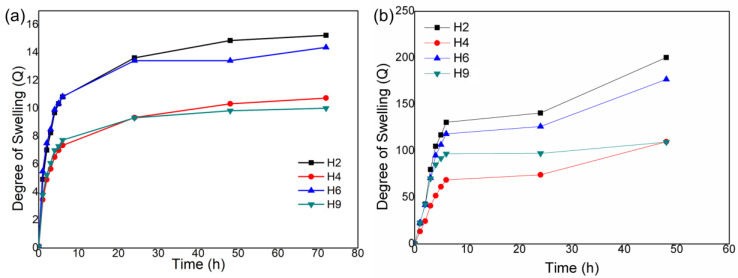
The degree of swelling as a function of time for the (**a**) non-hydrolysed and (**b**) hydrolysed H2, H4, and H9 hydrogels.

Figure 2a shows the degree of swelling in distilled water of the non-hydrolysed hydrogels as a function of hydration time. Figure 2b demonstrates that amide hydrolysis in an alkaline medium effectively increased the degree of swelling of the hydrogels more than 10-fold compared with the non-hydrolysed ones. Moreover, both non-hydrolysed and hydrolysed hydrogels showed a variable degree of swelling, probably due to the amount of AAm used in the formulation. The swelling equilibrium time was around 20 h for the non-hydrolysed hydrogels (Figure 2a) and >50 h for the hydrolysed hydrogels (Figure 2b). The presence of hydrophilic and hydrophobic groups is another factor linked to the absorption of water by the hydrogel, since hydrophilic groups interact with water, causing it to remain inside the hydrogel, unlike hydrophobic groups that prevent water from remaining inside the hydrogel. The synthesized hydrogels have amide -NH_2_ groups, hydroxyls OH, and also, the presence of silanol groups (SiO_2_) that can interact with water, causing it to remain inside the hydrogel.

Non-hydrolysed polyacrylamide hydrogels undergo changes from neutral hydrogels (before hydrolysis) to partially charged hydrogels (polyelectrolytes) after hydrolysis. As the neutral polyacrylamide hydrogel is immersed in an alkaline solution for a given time, the amide groups (-CONH_2_) are gradually transformed into ionized carboxyl groups due to hydrolysis. In other words, the amide groups in the polymeric chains in the network react with hydroxyl ions in an alkaline solution and are partially converted into the carboxylate and amine groups, [-CONH_2_ + NaOH → -COO^−^Na^+^ + NH_3_]. Therefore, the hydrolysed polyacrylamide/starch/SCBA in the H2 hydrogel increased its volume by almost 200-fold compared with the dry hydrogel, much higher than the 15-fold volume increase in the non-hydrolysed H2 hydrogel. This is due to the repulsion of negatively charged carboxylate groups in the hydrolysed hydrogel. Carboxylic groups interact more with water, thus leading to a higher degree of swelling [32,33,34,35].

PAAm hydrogels resulting from the polymerization of acrylamide present lower swelling values (Q) when the hydrogel presents a high degree of crosslinking, because the more crosslinked the polymer chain is, the less flexibility it will have, which makes it difficult for the expansion to occur and for water to diffuse into the interior of the hydrogel matrix. It can be noted that the hydrogels before the hydrolysis process presented swelling values of 10–15 g/g, which is related to the crosslinking of the polymer chain; that is, the polymer chain was not very flexible, which influenced the diffusion of water into the interior of the hydrogel. After hydrolysis, the formation of -COO^−^ groups occurred, which undergo electrostatic repulsion between themselves, being capable of increasing the expansion of the polymer chain and, thus, increasing the diffusion of water into the interior of the hydrogel. Consistently, the hydrolysed hydrogels presented a higher degree of swelling than the non-hydrolysed hydrogels. This fact arises from the interaction between the hydrogel that is favoured by hydrolysis. However, this interaction is not necessarily fast.

### 3.2. Swelling Behaviour of the Hydrogels at Different pHs and in Saline Solution

**Figure 3:**
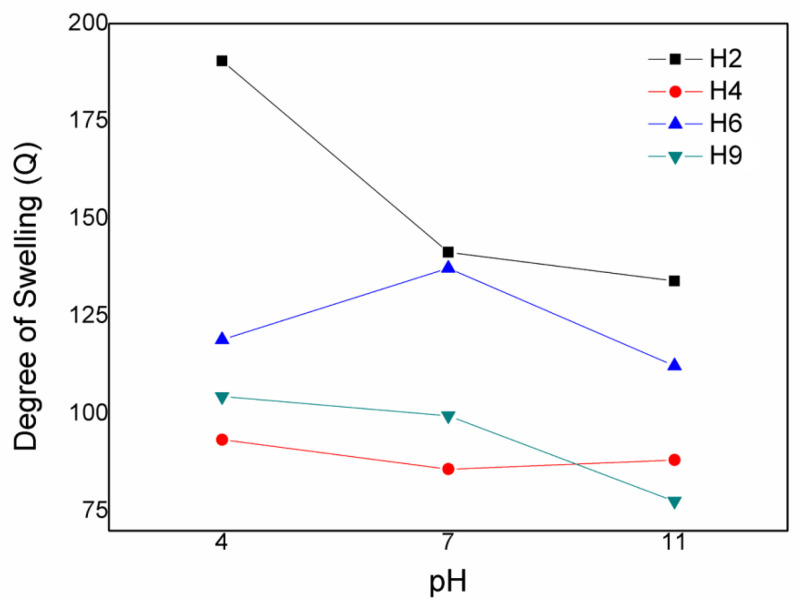
Degree of swelling of the hydrolysed H2, H4, H6, and H9 hydrogels in acidic (pH 4), neutral (pH 7.0), and alkaline (pH 11) media.

Figure 3 shows the degree of swelling of the hydrolysed hydrogels at different pHs. The hydrogels, except for the H2 hydrogel, swelled less in the acidic medium compared with the neutral and alkaline media. In this scenario, the carboxylate groups undergo protonation in acidic medium (pKa ≈ 4.5), and this minimises the anion–anion repulsive forces [36]. This fact may be related to the ionisation of the carboxyl groups present in the hydrogels. In this case, ionisation promotes the formation of carboxylate and through electrostatic repulsion between the carboxylate-containing chains, promoting the diffusion of water into 3D matrix to minimise the repulsive forces, leading to an expansion of the entire polymeric chain’s matrix [27]. The hydrogel becomes less hydrophilic when the pH is less than the pKa, which decreases the degree of swelling. Hydrogen bonds are possible in an acidic environment; that is, the degree of crosslinking of the chains increases. Therefore, the formation of hydrogen bonds hinders the movement of the chains in the hydrogel, forming a consistent hydrogel network when the pH is less than the pKa [27,37].

The degree of swelling increased for the H6 and H9 hydrogels, but not for the H2 and H4 hydrogels, at pH 7 compared with pH 4. The H4 hydrogel presented the smallest Q value at this pH. The H4 hydrogel contains more starch (approximately 3.5 g) than the H2 (1.0 g), H6 (1.0 g), and H9 (2.0 g) hydrogels. The higher amount of starch reduced the Q value at pH 7 due to increased mechanical crosslinking (entanglement) points in the polymeric network, which increased the crosslinking density of the hydrogel, resulting in a decrease in water diffusion in the hydrogel. This decrease may also be related to the fact that starch is partially filled in the hydrogel network; therefore, the number of hydrophilic groups is considerably smaller, and the Q value tends to decrease [38].

At pH 10, the Q value increased for the H4 hydrogel and decreased for the H2, H6, and H9 hydrogels. This phenomenon may be related to the ionisation of the carboxy groups present in the studied hydrogel formulations. Ionisation promotes electrostatic repulsion between the chains, promoting the diffusion of water into the 3D matrix to minimise the repulsive forces, thus expanding the entire polymeric chain matrix [27].

In summary, the H2 and H6 hydrogels presented the highest Q values and the H4 and H9 hydrogels presented the lowest Q values in all studied media. These data may be related to the starch content present in the gels, given that the H2 and H6 hydrogels have the lowest starch content, while the H4 and H9 hydrogels have the highest starch content. The data also indicate that the addition of SCBA does not influence the swelling process as much as starch: the H6 hydrogel has the highest SCBA content, but its Q values were higher than the H4 and H9 hydrogels, which have the lowest SCBA content.

**Figure 4:**
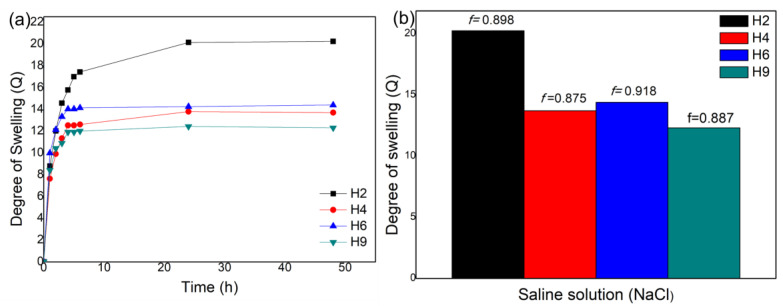
(**a**) A graph showing the degree of swelling graph as a function of time in saline solution for the non-hydrolysed H2, H4, H6, and H9 hydrogels; (**b**) the sensitivity factor (f) for the H2, H4, H6, and H9 hydrogels at equilibrium.

Due to the large number of ions present in the cement matrix, it is important to evaluate the performance of the degree of swelling in saline solution, which is representative of the cement medium. Figure 4a shows the Q values for the H2, H4, H6, and H9 hydrogels in saline solution. The H2 hydrogel presented the highest Q value, and the H9 hydrogel presented the lowest Q value. As shown in Figure 4b, the Q values in saline solution reduced sharply compared with acidic, neutral, and alkaline media, as denoted by the sensitivity factor (f), calculated with Equation (2):

f = 1 − [Qsal/Qwater](2)

where Qsal is the degree of swelling in saline solution, and Qwater in distilled water. Based on the f values, the saline effects are not as pronounced for the H9 and H4 hydrogels, as they are from the H2 and H6 hydrogels. The results indicate that the ash content does not affect the f value. Ash calcined at 600 °C was used in all the hydrogels: 10% for the H2 and H4 hydrogels, 30% for the H6 hydrogel, and 20% for the H9 hydrogel. The amount of SCBA in hydrogels and the ash granulometry should affect the swelling capacity. It was expected that, in high quantities, ash can fill the pores of the gels, making it difficult to increase Q. However, this behaviour was not observed for the solution, because the H6 hydrogel presented higher Q values than the H4 and H9 hydrogels.

These results corroborate findings from the literature: the presence of ions reduces the degree of swelling [24]. When the hydrogel is immersed in a saline solution (containing positive ions), interactions may occur between the hydroxyl groups present in the polymer chain and the sodium ions of NaCl, thus forming ionic pairs between the species [39,40]. In other words, there are electrostatic repulsions between the polymer chains of the hydrogel [22,41]. The contraction of polymer networks may also occur, contributing to reduce the hydrophilicity of the material.

### 3.3. Evaluation of Desorption of Water Absorbed by the Hydrolysed Hydrogels

**Figure 5:**
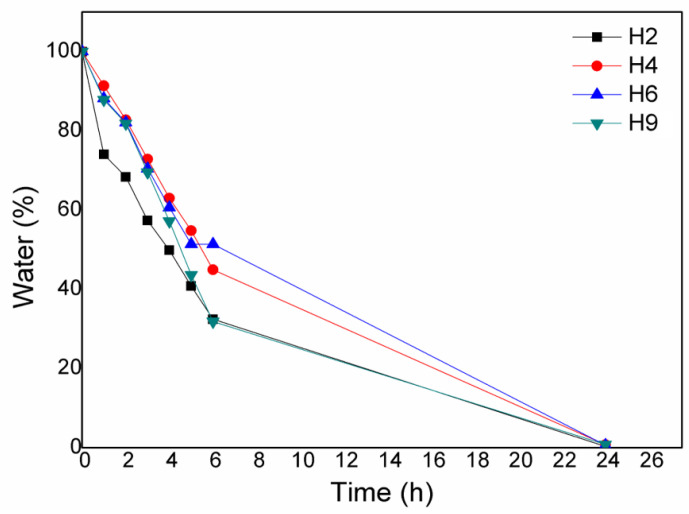
Water desorption from the H2, H4, H6, and H9 hydrogels over time.

Figure 5 shows the results of the water desorption assays. For this analysis, the swelling achieved during immersion for 24 h was considered to be the initial swelling. Therefore, the initial mass indicates 100% water present in the hydrogel matrix. Within 6 h, the H4 and H6 hydrogels released around 50% of the absorbed water, while the H2 and H9 hydrogels released 35% of the absorbed water. Furthermore, by 24 h, each hydrogel had released around 95% of the absorbed water, with no difference between the hydrogels. The desorption test was carried out in water, but the results are expected to be different in mortar. Therefore, if the hydrogel releases all the water retained in the polymeric matrix within 24 h, then the hydrogel is not completely effective, because mortar cures over a period of more than 28 days [4].

### 3.4. Morphology of the Hydrogels

**Figure 6:**
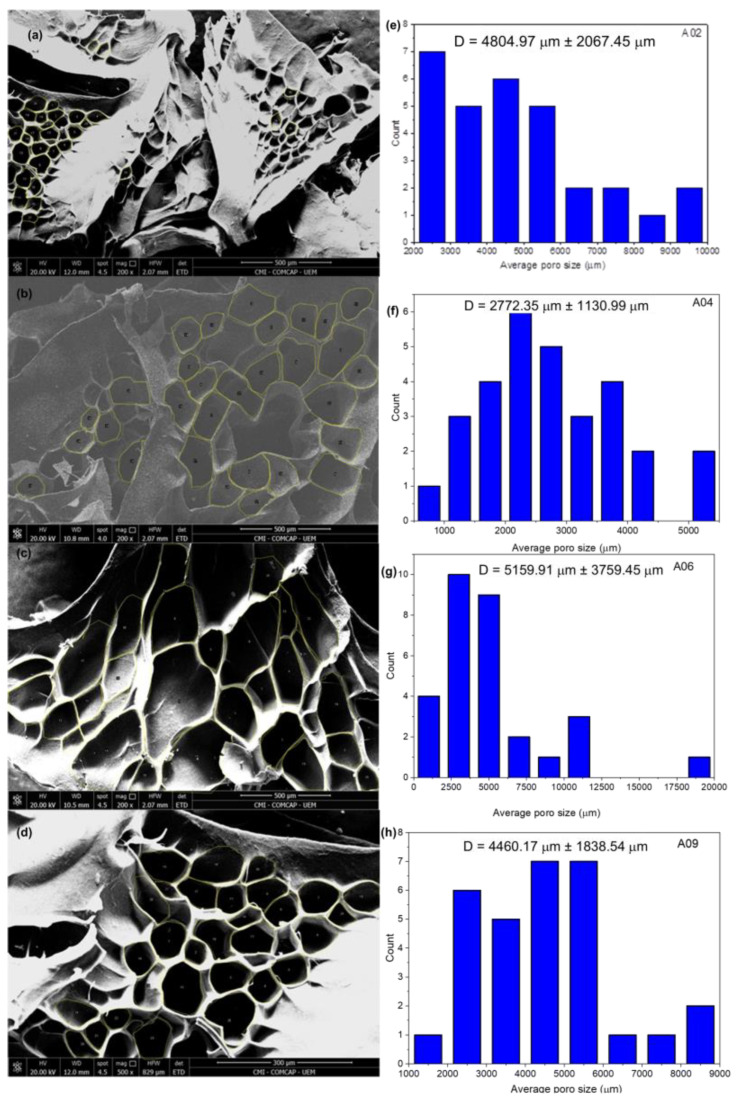
Scanning electrons micrographs (200× magnification) and the pore distribution of the hydrolysed (**a**) and (**e**) H2, (**b**) and (**f**) H4, (**c**) and (**g**) H6, and (**d**) and (**h**) H9 hydrogels.

Figure 6a–d shows representative scanning electron micrographs of the hydrolysed H2, H4, H6, and H9 hydrogels. They show that the hydrogels presented porous surfaces, as pores in irregular shapes and sizes are distributed throughout the polymer matrix, thus indicating that the addition of SCBA did not prevent the formation of pores in the hydrogels. Figure 6e–h shows the average pore size of the hydrolysed H2, H4, H6, and H9 hydrogels: 4804.97 ± 2067.45, 2772.35 ± 1130.99, 5159.91 ± 3759.45, and 4460.17 ± 1838.54 µm, respectively. It can be noted that the presence of SCBA can influence the formation of pores, since the H6 hydrogel presented larger pores than the others, and its formulation has the highest SCBA content. The H6 hydrogel has a larger pore size compared with the other hydrogels, a surprising finding given that it did not have the highest Q value; this may be related to a low starch content (1.0 g) compared with the H4 and H9 hydrogels (3.0 g and 2.0 g, respectively), thus indicating that the interactions between the hydrophilic groups present in the polymer matrix favour swelling more than the hydrogel pore size, considering that the H4 and H9 hydrogels have smaller pore sizes and higher Q values than the H6 hydrogel.

### 3.5. FTIR of the Hydrogels and XRD of SCBA

**Figure 7:**
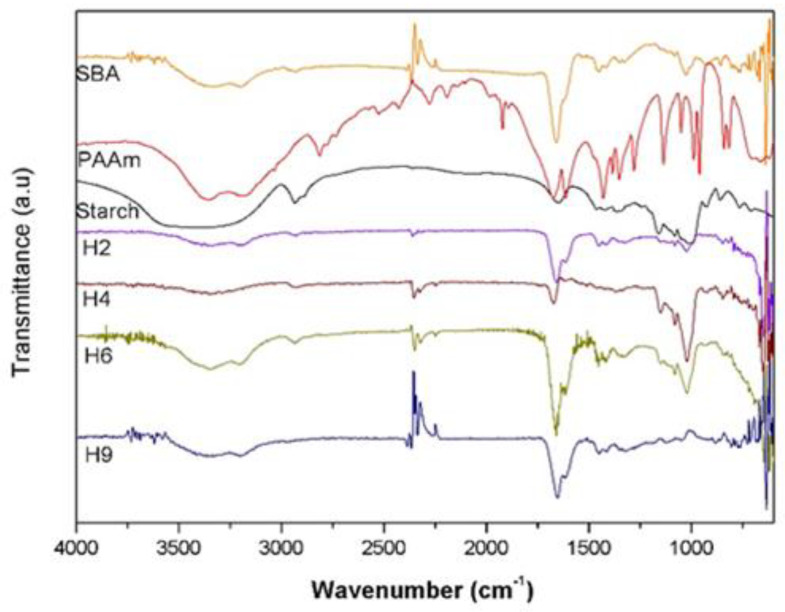
The attenuated total reflectance Fourier transform infrared spectra of sugarcane bagasse ash (SCBA), crosslinked polyacrylamide (PAAm), starch, and the hydrolysed H2, H4, H6, and H9 hydrogels.

The FTIR spectra of SCBA, PAAm, starch, and the hydrolysed H2, H4, H6, and H9 hydrogels are shown in Figure 7. For SCBA, the bands at 3392 and 1664 cm^−1^ are attributed to the O-H deformation of the silanol group (-SiOH); the bands at 1043 and 667 cm^−1^ are attributed, respectively, to the symmetric stretching and deformation of Si-O-Si [40,42]. For PAAm (formed from the polymerization of acrylamide (Amm)), the bands at 3357 and 3195 cm^−1^ refer to the axial deformation of the N-H bond, and the bands at 1676 cm^−1^ and 1605 cm^−1^ are attributed to the stretching vibration of the C=O groups and the N-H stretching vibration of the amide groups, respectively, belonging to the PAAm molecule [43,44]. For starch, the band at 3435 cm^−1^ refers to the O–H stretching vibration of the hydroxyl group present in the starch molecule. The carbon chain present in the starch molecule that generates the bands observed in 2943 cm^−1^ is due to the C–H stretching vibration; the bands at 1647, 1458, and 1353 cm^−1^ are related to the C–OH bending; and the bands at 1147, 1076, and 980 cm^−1^ correspond to the C–O stretching vibration [45].

It can be noted that the spectra of the hydrolysed hydrogels H2, H4, H6, and H9 are similar and have bands characteristic of both AAm and starch. In the spectrum of the hydrogels, a broad band can be noted at 3500–3100 cm^−1^ that is related to the overlap of the stretching band of the starch hydroxyl group (-OH) with the stretching band of the N-H bond of the amide present in the polyacrylamide, formed by the polymerisation of the acrylamide monomer. In addition, the band around 1700 cm^−1^ corresponds to carboxyl (C=O) also present in acrylamide. The hydrogels H2 and H4 showed a decrease in the band corresponding to carboxyl, possibly due to the hydrolysis reaction, since, in this process, the amide groups (-CONH_2_) are converted to carboxylate groups (-COO^−^Na^+^). It can also be noted that the shift of the N-H vibration band of the amide at 1605 cm^−1^ to 1461 cm^−1^ in the hydrogels indicates that crosslinking reactions occurred [24].

**Figure 8:**
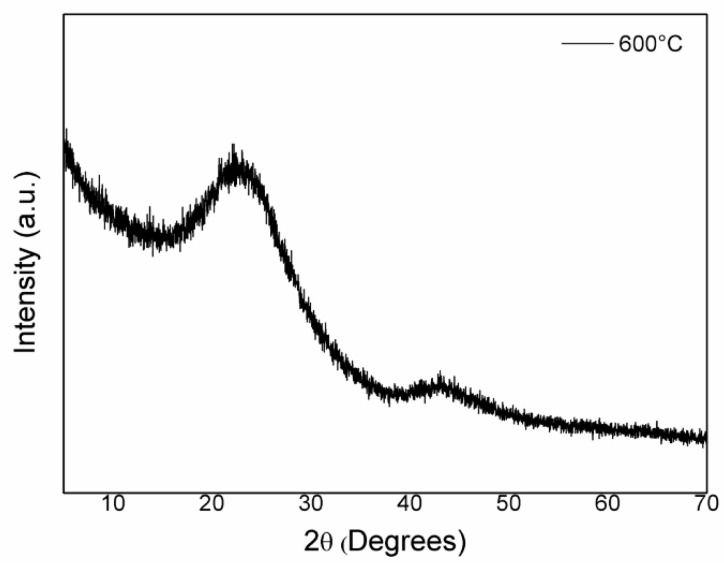
The X-ray diffraction profile of sugarcane bagasse ash obtained by calcination at 600 °C for 2 h.

The SCBA was obtained through the calcination of SBA at 600 °C. This material had a higher level of pozzolanic activity [23]. Calcination at 600 °C eliminates, by burning, almost all the carbon present in the sample structure [23,24]. The X-ray diffractogram presented in Figure 8 showed an amorphous halo originating from the silica between 2*θ* of 10° and 50°. Moreover, there are no crystallinity peaks. Therefore, its reactivity is better, and, consequently, it has better applicability to cementitious media [46].

### 3.6. FTIR, XRD, and TGA of the Cement Pastes 

**Figure 9:**
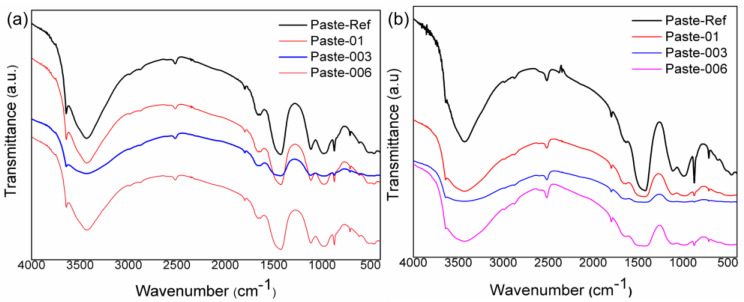
The Fourier transform infrared spectra of cement pastes aged for 7 days (**a**) and 28 days (**b**).

Figure 9 shows the FTIR spectra of the aged cement pastes. After ageing for 7 and 28 days, there were no changes in the absorption bands in relation to the reference cement paste. This result indicates that the addition of hydrogel into the concrete matrix did not significantly change the composition of the cement pastes. The bands observed at 3629, 3638, and 3658 cm^−1^ correspond to the hydroxyl group (-OH) present in Ca(OH)_2_; this formation results from the hydration reactions of the compounds C_3_S, C_2_S, and C-S-H [45]. The bands observed at 1452 and 732 cm^−1^ refer to the -CO bond of the carbonate groups. The band at 991 cm^−1^ is attributed to the asymmetric stretching of the Si-O vibrations of the SiO_4_ tetrahedron of the silicate phase present on C_3_S and C_2_S. In all samples, the vibrations at 713 and 870 cm^−1^ indicate the presence of calcite (CaCO_3_) [47].

**Figure 10:**
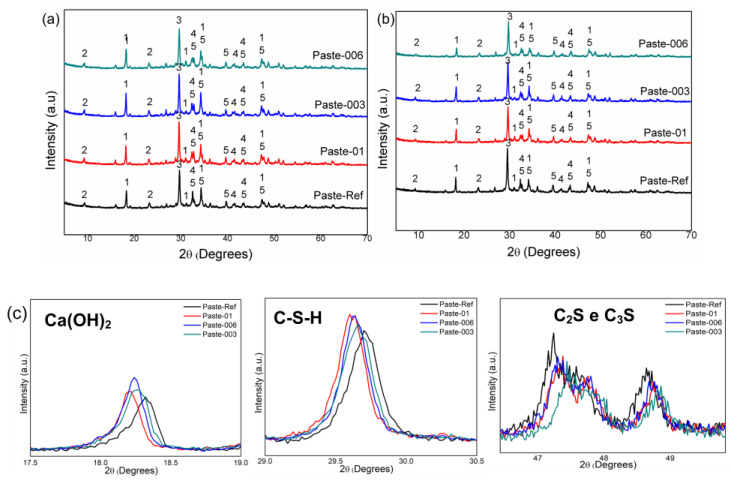
Comparison of the X-ray diffractograms of the cement pastes after ageing for (**a**) 7 and (**b**) 28 days and (**c**) broadening of the (CaOH)_2_, C-S-H, and C_2_S and C_3_S peaks.

The hydrolysed H2 hydrogel was used to produce the cement pastes due to its degree of swelling from the water absorption and desorption tests (see Table 2 and Figure 2 and Figure 3). Figure 10 shows the XRD profiles obtained in cement pastes after ageing for 7 and 28 days from the reference paste (Paste-ref) and pastes with 0.06%, 0.03%, and 0.1% hydrogel by weight. The pastes with hydrogel and the reference paste (without gel addition) present similar X-ray diffractograms, indicating that the addition of hydrogel did not modify the profiles of the cement paste matrix during curing for 7 and 28 days.

The X-ray diffractograms were analysed to identify the anhydrous and hydrated crystalline phases in the pastes: (1) portlandite (Ca(OH)_2_), (2) ettringite, (3) C-S-H, (4) dicalcium silicate (C_2_S), and (5) tricalcium silicate (C_3_S). In general, portlandite was most detectable, with 2θ peaks of 18°, 31°, 34°, and 46° after 7 and 28 days of curing. Portland cement basically consists of C_2_S and C_3_S. During the hydration reactions, C_2_S and C_3_S are transformed into Ca(OH)_2_ and C-S-H. In other words, the presence (or absence) of C_2_S, C_3_S, Ca(OH)_2_, and C-S-H can be noted in the X-ray diffractogram patterns, so it may be indicative of the formation (or not) of such compounds during ageing. The addition of hydrogel to the cement matrix can delay the hydration of the concrete, as it adheres to the particles, thus preventing heat conduction between the cement particles [48,49].

Figure 10c shows that, for all cement pastes containing hydrogel, the peak at 2θ = 18° is more intense than the reference paste for both curing ages studied, i.e., there was greater production of Ca(OH)_2_, thus indicating better hydration of the medium. Furthermore, it can also be noted that the peak at 2θ = 29° (referring to C-S-H) is also more intense for the pastes containing hydrogel, indicating more formation of C-S-H. It is also possible to note that the peaks referring to C_2_S and C_3_S (2θ = 39° and 41°) are more intense for the reference paste (without the addition of hydrogel), indicating that this sample contains a greater presence of non-hydrated products, while the samples with hydrogel have smaller peaks of these compounds, indicating that hydration was more efficient [30].

**Figure 11:**
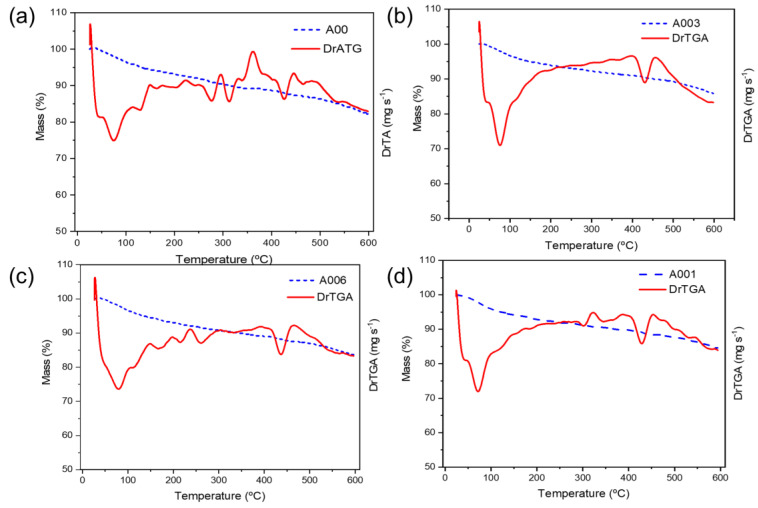
Thermogravimetric (TG)/derivative thermogravimetric (DTG) analysis of Portland cement pastes aged for 28 days: (**a**) reference paste, (**b**) with 0.1% hydrogel by weight, (**c**) with 0.03% hydrogel by weight, and (**d**) with 0.06% hydrogel by weight.

Figure 11 shows the thermogravimetric (TG)/first-derivative thermogravimetric (DTG) curves of the hydrated cement pastes, after ageing for 28 days. The TG/DTG profiles demonstrate the reactions that occurred in the hydrated cement pastes when subjected to a continuous increase in temperature. The TG/DTG curves of the pastes present a peak close to 100 °C; this peak is associated with the release of water and dehydration of C-S-H and ettringite. Due to this overlap, it is not possible to calculate the C-S-H dose accurately. However, another peak around 440 °C corresponds to the dehydration of Ca(OH)_2_ [6,47,49]. At this temperature, it can be noted that the cement paste without hydrogel (Figure 11a) presented a mass loss of 10%, while those with the addition of hydrogel presented a mass loss of 15%, thus indicating a greater presence of Ca(OH)_2_ [30].

### 3.7. Compressive Strength Tests of the Mortars

**Figure 12:**
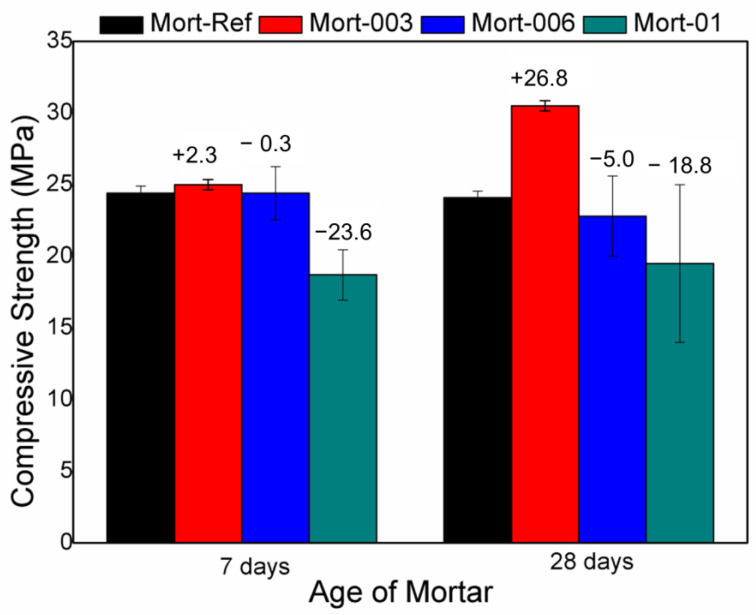
Compressive strength test for the mortars aged for 7 and 28 days. Mort-ref, reference mortar; Mort-003, mortar with 0.03% hydrogel by weight; Mort-006, mortar containing 0.06% hydrogel by weight; and Mort-01, mortar containing 0.1% hydrogel by weight.

Figure 12 shows the compressive test results for the mortars containing the hydrolysed H2 hydrogel (0.1%, 0.03%, and 0.06% by weight: Mort-01, Mort-003, and Mort-006, respectively). After ageing for 7 days, only Mort-003 presented an increase in resistance relative to the reference mortar (Mort-Ref); the resistance of Mort-006 and Mort-001 decreased relative to Mort-Ref. After ageing for 28 days, similar behaviour was exhibited: an increase for Mort-003 and a reduction for Mort-006 and Mort-01 relative to Mort-Ref.

The addition of 0.03% hydrogel proved to be effective in increasing the mechanical resistance of the mortar. This may be related to the controlled release of water in the cementitious matrix, favouring the curing process [47,50]. This increase can be attributed to the presence of swollen hydrogel in the formulation, because the added hydrolysed H2 hydrogel was swollen at equilibrium. So, in the preparation of the mortar, no additional water was added to the specimens, differently from the cement pastes prepared without hydrogel. The hydrogel released water during ageing, favouring effective curing. The addition of 0.1% and 0.06% hydrogel by weight was unfavourable in terms of resistance. After ageing for 28 days, the discrepancy in the resistance was considerable. The reduction in the strength of Mort-01 and Mort-006 due to the addition of hydrogel can be caused by the formation of voids in the mortar microstructure, precisely because, as water is released by the swollen hydrogels in the cement matrix, there is a reduction in the polymer, causing the formation of voids in the microstructure. The differences in the compressive test results are related to different factors, such as climate and the moulding of the test specimens [51,52,53]. The compressive test results showed that the developed hydrogels have the capacity to act as an internal curing agent, providing better hydration for the cementitious medium and, consequently, greater mechanical resistance. Furthermore, only a small amount of the hydrolysed H2 hydrogel (0.03% by weight) is necessary to improve the resistance of the material, while higher levels can reduce resistance.

The fact that Mort-01 and Mort-006 presented lower resistance compared with Mort-Ref does not mean that the hydrolysed H2 hydrogel cannot act as an internal curing agent. Instead, it can be said that high levels of hydrogel end up releasing a greater amount of water than is necessary to act in the hydration process. This excess water harms the resistance of the material, because a cementitious medium must have a proper balance of its constituent materials [54,55].

In the literature, there are no discussions about the interaction between the hydrogel and the cementitious medium. However, what can occur is the interaction between the polymer matrix and the ions formed in the cement hydration process. As has already been discussed, the presence of ions reduces the absorption of water by the hydrogel. For this reason, in our research, the hydrogels were already swollen when added, so that they could absorb and release the greatest possible amount of water, without being influenced by the environment in which they were inserted.

In addition to hydrogels, another material that is widely studied for use as an internal curing agent is prewetted lightweight aggregates (LWAs), which, like hydrogels, improve relative humidity in the environment by releasing water [56]. However, despite being effective in reducing autogenous shrinkage, the use of LWAs caused a reduction in the mechanical strength of the material, due to the formation of large pores and the increase in the porosity of the material [56,57,58]. Furthermore, the use of LWAs implies the reduction in fine and coarse aggregates [56,57], which can also be a factor responsible for the reduction of mechanical resistance. Thus, the results obtained can indicate that hydrogels have an advantage over LWAs, as they are capable of reducing autogenous shrinkage and increasing mechanical resistance without the need to reduce another component of the medium.

The water contained in the inner part of the hydrogel is more difficult to be lost through evaporation, as it interacts with the hydrophilic groups present in the polymer matrix, when compared with the free water in the cement pores. In addition, the water absorbed by the hydrogel will be desorbed directly into the cementitious medium, since the hydrogel will be surrounded by the mass formed by the cement hydration process, thus creating another barrier to water loss through evaporation.

The compressive test data were subjected to analysis of variance (ANOVA) to evaluate the statistical significance of the main factors: age (7 and 28 days) and the hydrolysed hydrogel content (0.03%, 0.06%, and 0.01% by weight). Table 3 shows the age, hydrolysed H2 hydrogel content as well as the age × hydrolysed hydrogel content interaction. The age, hydrolysed hydrogel content, and age × hydrogel content interaction were significant. Hence, the amount of hydrolysed hydrogel in the cementitious material (e.g., mortar in this study) and the curing time are highly relevant to the mechanical properties of the cementitious material.

## 4. Conclusions

The synthesis of hydrogels from crosslinked PAAm, starch, and SCBA was successful; however, the hydrogels presented low Q values (between 10–15 g/g). After the hydrolysis process, an increase of more than 10 times in the Q value (110–200 g/g) was observed, thus producing superabsorbent hydrogels. SEM images showed that the addition of SCBA did not influence the formation of pores, and SCBA particles were not deposited on the surface of the hydrogel.

FTIR and XRD analyses of the cement pastes indicate that the addition of the hydrogel to the cementitious medium does not alter the composition of the cement pastes, showing that the hydrogel can be added to the cementitious medium without damaging the main characteristics of product. When analysing the mechanical properties, it can be noted that the addition of the 0.03% content was able to increase the mechanical strength in the curing period of 7 and 28 days, indicating that the hydrogel acted as an internal curing agent, improving hydration and, thus, improving the mechanical strength. However, when contents higher than 0.03% (0.06% and 0.1%) were added, a reduction in mechanical strength occurred in up to 26.8% at 28 days of curing, as compared with reference (without hydrogel). This does not mean that the hydrogel did not act as an internal curing agent and is related to the formation of voids after the release of water by the hydrogel, which negatively influences the strength of the material.

In addition to what was reported above, to the best of our knowledge, this is the first time that a hydrogel containing crosslinked PAAm, starch, and SCBA has been used as a curing agent for mortars and cement pastes. In addition to the technological use of hydrogel as a curing agent, this approach is environmentally friendly, because it uses a natural product (starch) and SCBA, a byproduct of the sugarcane industry. This research paves the way for the development of new research using hydrogels containing crosslinked PAAm, starch, and SCBA with application in the cementitious medium (using the hydrogels that have not yet been applied in the cementitious medium) and for new applications that require materials that can absorb and release large amounts of water.

## Figures and Tables

**Table 1:** Factors and their levels used in the development of factorial design.

Factors (g)	Lower Level (−)	Central Point (0)	Upper Level (+)
AAm	1.5	2.5	3.5
Starch	1.0	2.0	3.0
SCBA	0.10	0.20	0.30

**Table 2:** Complete factorial design matrix with all formulations used for hydrogel synthesis.

Run	AAm Content	Starch Content	SCBA Content
H1	1.5	1.0	0.10
H2	3.5	1.0	0.10
H3	1.5	3.0	0.10
H4	3.5	3.0	0.10
H5	1.5	1.0	0.30
H6	3.5	1.0	0.30
H7	1.5	3.0	0.30
H8	3.5	3.0	0.30
H9a	2.5	2.0	0.20
H9b	2.5	2.0	0.20
H9c	2.5	2.0	0.20

**Table 3:** Analysis of variance of the compressive strength test data of the mortars, considering the main factors age and the hydrolysed hydrogel content.

Effect	SQ *	DF **	MQ ***	F	*p*-Value	Result
Age	$2.83\;\times \;10^{14}$	11	$2.56\;\times \;10^{13}$	3.8	$2.3\;\times \;10^{-3}$	Significant
Hydrogel	$2.28\;\times \;10^{14}$	6	$7.60\;\times \;10^{13}$	11.2	$4.0\;\times \;10^{-4}$	Significant
Residue	$1.10\;\times \;10^{14}$	-	$6.77\;\times \;10^{12}$	-	-	-

* SQ, quadratic sum; ** DF, degrees of freedom; *** MQ, mean of squares.

## Data Availability

The original contributions presented in the study are included in the article, further inquiries can be directed to the corresponding author.
